# Supplementary material for: Associations between prenatal caffeine exposure and child development: Longitudinal results from the Adolescent Brain Cognitive Development (ABCD) Study
Source: medRxiv. 2024 Jun 19:2024.06.18.24309117. Preprint. [Version 1] doi: 10.1101/2024.06.18.24309117 (PMC11213099; doi:10.1101/2024.06.18.24309117)
Supplement: Supplement 2 [file media-2.pdf]

Supplement 2: Results

Longitudinal Outcomes of Exposure Without Covariates

Before covariate adjustment, any level of prenatal caffeine exposure was associated with social, attention, thought, internalizing, externalizing, and sleep problems (all $\beta > 0.13$; all FDR-corrected $P < .008$; **eTable 1**). Daily exposure was associated with higher scores on these scales as well, in addition to increased PLEs (all $\beta > 0.17$; all FDR-corrected $P < .04$; **eTable 1**).

Outcomes of Exposure Without Covariates in the Daily Caffeine Subset

Longitudinal analysis without covariate adjustment in all daily users showed that multiple-cup daily caffeine exposure was associated with increased scores on all five CBCL subscales, as well as increased PLEs and sleep problems ($\beta > 0.11$, FDR-corrected $P < .02$; **eTable 2**). Any daily exposure as compared to controls was also marginally/nominally significant for an increase in attention problems (($\beta = 0.1$; $P = .05$; FDR-corrected $P = 0.2$; **eTable 2**).

Analyses with Exclusions.

Excluding children for uncommon substance use. Results were largely consistent with those reported using the full sample before FDR correction (**eTables 3 and 7**), the only exceptions being that children exposed to multiple cups of caffeine daily had significantly higher social and thought problems than those exposed to one cup or less daily (all $\beta > 0.154$, all $p < 0.05$), and the significant difference in attention problems between those exposed daily and those exposed less frequently was reduced to a trend ($\beta = 0.142$, $p = 0.071$).

Excluding children exposed to uncommon substances during pregnancy. Results were consistent with those reported using the full sample (**eTables 4 and 8**).

Excluding children born prematurely. Results were consistent with those reported using the full sample (**eTables 5 and 9**).

Excluding children whose parent/caregiver report was not their biological mother. As described in **eTables 6 and 10**, results were consistent with those reported using the full sample.
