# Supplementary material for: Associations between prenatal caffeine exposure and child development: Longitudinal results from the Adolescent Brain Cognitive Development (ABCD) Study
Source: medRxiv. 2024 Jun 19:2024.06.18.24309117. Preprint. [Version 1] doi: 10.1101/2024.06.18.24309117 (PMC11213099; doi:10.1101/2024.06.18.24309117)
Supplement: Supplement 3 [file media-3.pdf]

Figure S1. Distribution of Prenatal Caffeine Exposure Across Cohort.

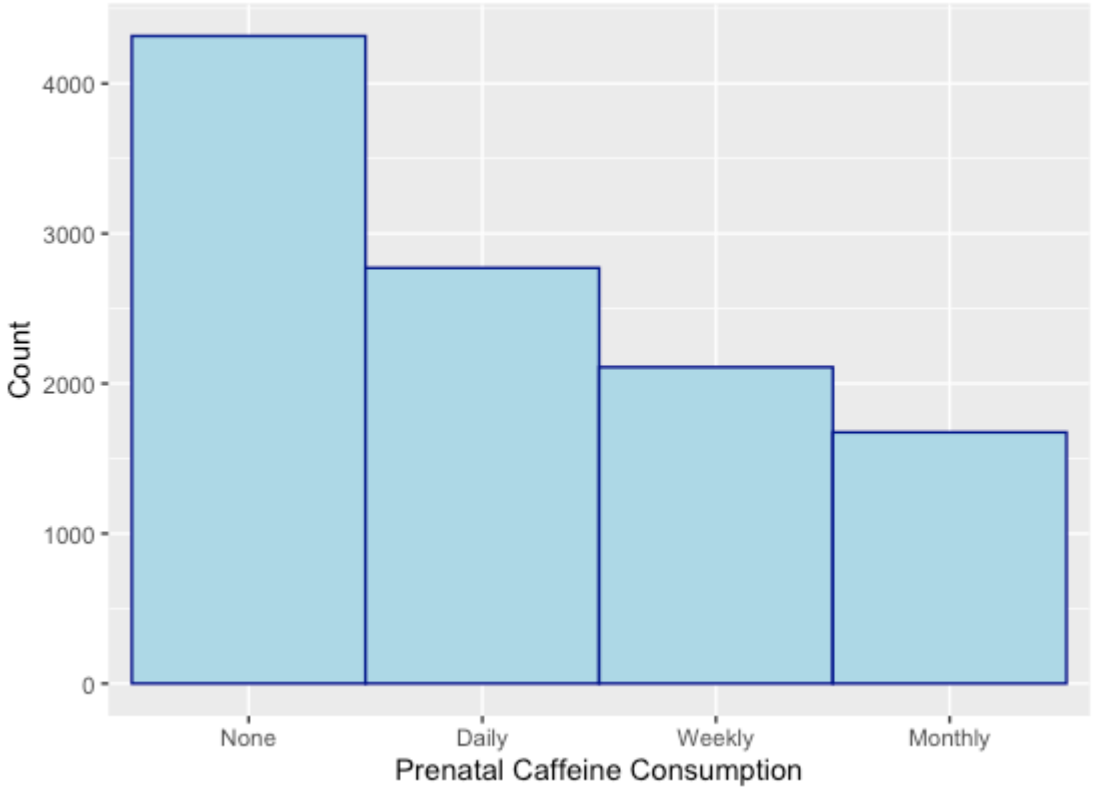

Figure S1 Note. Prenatal caffeine consumption was coded within the ABCD dataset as a categorical variable with four levels: none, daily, weekly, and monthly exposure. Maternal caffeine consumption during pregnancy was a retrospective self-report taken at the baseline timepoint. Exact count in each group is presented in **Table 1**.
