# Supplementary material for: Associations between prenatal caffeine exposure and child development: Longitudinal results from the Adolescent Brain Cognitive Development (ABCD) Study
Source: medRxiv. 2024 Jun 19:2024.06.18.24309117. Preprint. [Version 1] doi: 10.1101/2024.06.18.24309117 (PMC11213099; doi:10.1101/2024.06.18.24309117)
Supplement: Supplement 4 [file media-4.pdf]

Figure S2. Age Interaction Plot for Prenatal Caffeine Exposure and Attention Problems.

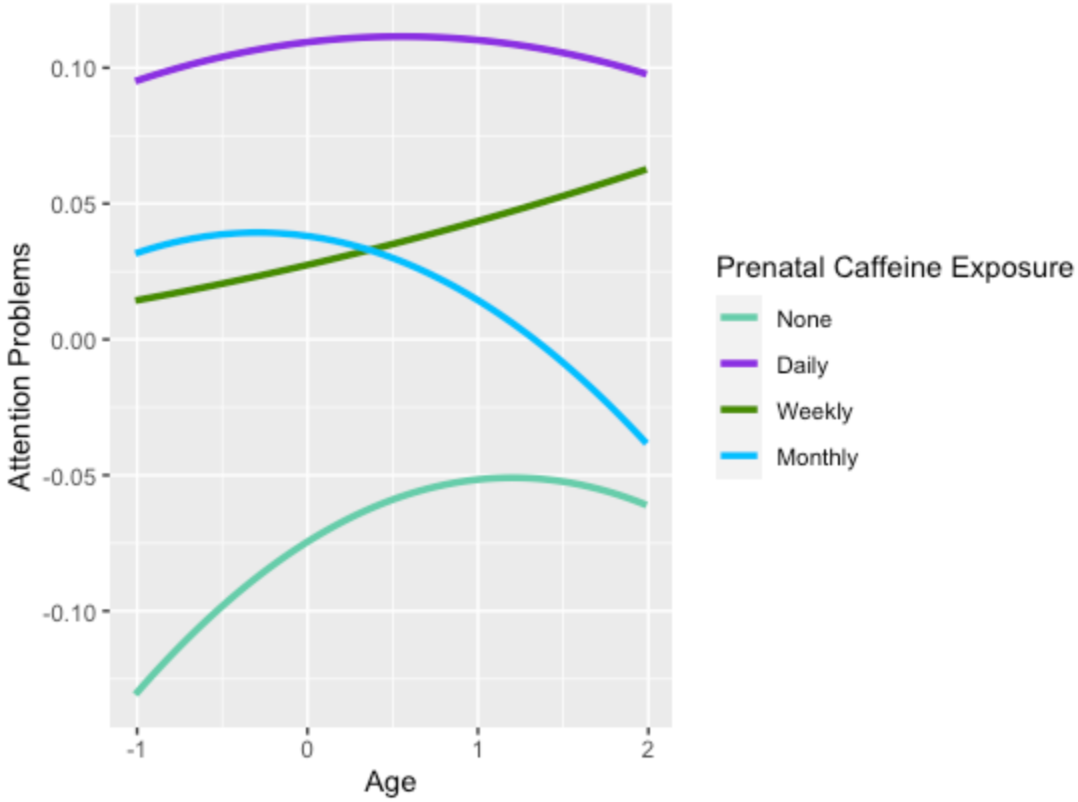

Figure S2 Note. Age effects were determined using a linear mixed-effects model. Age and age squared values were derived from the ABCD interview age variable. Attention problems were derived from the Child Behavior Checklist. Both axes were log-transformed and normalized. Prenatal caffeine consumption was coded within the ABCD dataset as a four-level categorical variable.
