# Supplementary material for: Associations between prenatal caffeine exposure and child development: Longitudinal results from the Adolescent Brain Cognitive Development (ABCD) Study
Source: medRxiv. 2024 Jun 19:2024.06.18.24309117. Preprint. [Version 1] doi: 10.1101/2024.06.18.24309117 (PMC11213099; doi:10.1101/2024.06.18.24309117)
Supplement: Supplement 5 [file media-5.pdf]

Table S1. Prenatal Caffeine Exposure and Child Outcomes Omnibus Testing

Outcome (n = 8969)	χ^2	*P* value	FDR-corrected P value
Psychotic-like experiences	7.19	0.07	0.19
Internalizing per CBCL	2.99	0.39	0.53
Externalizing per CBCL	10.03	**0.02**	0.08
Attention per CBCL	2.81	0.42	0.53
Thought per CBCL	0.22	0.98	0.98
Social per CBCL	2.57	0.46	0.53
Body mass index[b]	12.57	**5.67E-03**	**0.045**
Total sleep problems	5.98	0.11	0.22

Table S1 Note. Log-likelihood ratio tests were used to analyze the associations between prenatal caffeine exposure (a factor variable with 4 mutually exclusive groups: no, daily, weekly, and monthly exposure) and each outcome, nesting data by research site and family identification. All covariates listed in the methods were included in the analysis. Psychotic-like experiences were assessed with the Prodromal Questionnaire Brief-Report Child Version and sleep problems from the Parent Sleep Disturbance Scale for Children.

[a]Due to high missingness of anthropometric data at follow-up waves, BMI was only analyzed as an outcome at the baseline wave.
