# Supplementary material for: Associations between prenatal caffeine exposure and child development: Longitudinal results from the Adolescent Brain Cognitive Development (ABCD) Study
Source: medRxiv. 2024 Jun 19:2024.06.18.24309117. Preprint. [Version 1] doi: 10.1101/2024.06.18.24309117 (PMC11213099; doi:10.1101/2024.06.18.24309117)
Supplement: Supplement 6 [file media-6.pdf]

Table S2. Age Interaction Effects on Associations Between Prenatal Caffeine Exposure and Outcomes of Interest

**Outcome**	χ^2	***P* value**	**FDR-corrected P value**
Psychotic-like experiences	2.47	0.87	0.87
Internalizing per CBCL	9.64	0.14	0.25
Externalizing per CBCL	12.32	0.06	0.19
Attention per CBCL	22.82	**8.58E-04**	**0.01**
Thought per CBCL	4.14	0.66	0.77
Social per CBCL	11.02	0.09	0.21
Total sleep problems	6.82	0.34	0.47

Table S2 Note. Prenatal caffeine exposure was coded as a 4-level categorical variable in the ABCD dataset (daily, weekly, monthly, and no exposure). Two separate linear mixed-effects models were used to analyze age effects: 1) interview age included as a covariate; and 2) age x caffeine and age^2 x caffeine included as interaction terms. These models were compared using log-likelihood ratio tests to determine the outcomes with significant age interactions.
