# Supplementary material for: Associations between prenatal caffeine exposure and child development: Longitudinal results from the Adolescent Brain Cognitive Development (ABCD) Study
Source: medRxiv. 2024 Jun 19:2024.06.18.24309117. Preprint. [Version 1] doi: 10.1101/2024.06.18.24309117 (PMC11213099; doi:10.1101/2024.06.18.24309117)
Supplement: Supplement 7 [file media-7.pdf]

Table S3. Post Hoc Analysis of Age Effects on Attention Problems and Prenatal Caffeine Exposure Associations

	**Age**			**Age²**		
**Contrast**	**β**	***P* value**	**FDR-corrected P value**	**β**	***P* value**	**FDR-corrected P value**
Daily vs. no exposure	-0.01	0.41	0.55	-0.02	**0.02**	0.06
Weekly vs. no exposure	0.02	**0.04**	0.08	-0.01	0.45	0.52
Monthly vs. no exposure	-0.01	0.54	0.61	-0.04	**4.38E-04**	**0.004**
Daily vs. weekly	-0.03	**0.01**	0.07	-0.01	0.2	0.27
Daily vs. monthly	-0.001	0.93	0.93	0.02	0.16	0.25
Weekly vs. monthly	0.03	**0.02**	0.07	0.03	**0.01**	0.05
Any caffeine exposure vs. no exposure	0.06	**0.02**	0.07	-0.04	0.07	0.15
Daily caffeine exposure vs. all lower exposure	-0.05	0.14	0.23	0.01	0.74	0.74

Table S3 Note. Prenatal caffeine exposure was coded as a 4-level categorical variable in the ABCD dataset (daily, weekly, monthly, and no exposure). Two separate linear mixed-effects models were used to analyze age effects: 1) interview age included as a covariate; and 2) age x caffeine and age^2 x caffeine included as interaction terms. β coefficients are standardized and are from regressions accounting for covariates (see methods).
