# Supplementary material for: Associations between prenatal caffeine exposure and child development: Longitudinal results from the Adolescent Brain Cognitive Development (ABCD) Study
Source: medRxiv. 2024 Jun 19:2024.06.18.24309117. Preprint. [Version 1] doi: 10.1101/2024.06.18.24309117 (PMC11213099; doi:10.1101/2024.06.18.24309117)
Supplement: Supplement 8 [file media-8.pdf]

Table S4. Associations of All Outcomes with All Prenatal Caffeine Exposure Levels

Outcome (n = 8991)	Contrast	β	*P* value	FDR-corrected *p* value
**Psychotic-like experiences**	Daily caffeine exposure vs no exposure	0.04	0.07	0.21
	Weekly caffeine exposure vs no exposure	-0.02	0.31	0.49
	Monthly caffeine exposure vs no exposure	0.002	0.93	0.93
	Daily caffeine exposure vs weekly exposure	0.06	**0.01**	0.09
	Daily caffeine exposure vs monthly exposure	0.04	0.15	0.34
	Weekly caffeine exposure vs monthly exposure	-0.03	0.75	0.49
	Any caffeine exposure vs no exposure	-0.05	0.38	0.49
	Daily caffeine exposure vs all lower exposure	0.14	**0.02**	0.13
**Internalizing per CBCL**	Daily caffeine exposure vs no exposure	-0.003	0.89	0.89
	Weekly caffeine exposure vs no exposure	0.01	0.67	0.89
	Monthly caffeine exposure vs no exposure	0.04	0.12	0.54

	Daily caffeine exposure vs weekly exposure	-0.01	0.6	0.89
	Daily caffeine exposure vs monthly exposure	-0.04	0.12	0.54
	Weekly caffeine exposure vs monthly exposure	-0.03	0.31	0.7
	Any caffeine exposure vs no exposure	-0.02	0.86	0.89
	Daily caffeine exposure vs all lower exposure	-0.05	0.71	0.89
**Externalizing per CBCL**	Daily caffeine exposure vs no exposure	0.07	**0.003**	**0.01**
	Weekly caffeine exposure vs no exposure	0.02	0.5	0.72
	Monthly caffeine exposure vs no exposure	0.01	0.75	0.77
	Daily caffeine exposure vs weekly exposure	0.05	**0.04**	0.08
	Daily caffeine exposure vs monthly exposure	0.06	**0.03**	0.06
	Weekly caffeine exposure vs monthly exposure	0.01	0.77	0.77

	Any caffeine exposure vs no exposure	-0.07	0.56	0.72
	Daily caffeine exposure vs all lower exposure	0.39	**0.002**	**0.01**
**Attention per CBCL**	Daily caffeine exposure vs no exposure	0.03	0.15	0.51
	Weekly caffeine exposure vs no exposure	0.02	0.54	0.74
	Monthly caffeine exposure vs no exposure	0.03	0.23	0.51
	Daily caffeine exposure vs weekly exposure	0.02	0.48	0.74
	Daily caffeine exposure vs monthly exposure	0.003	0.93	0.93
	Weekly caffeine exposure vs monthly exposure	-0.02	0.58	0.74
	Any caffeine exposure vs no exposure	0.03	0.67	0.75
	Daily caffeine exposure vs all lower exposure	0.1	0.21	0.51
**Thought per CBCL**	Daily caffeine exposure vs no exposure	-0.004	0.88	0.92

	Weekly caffeine exposure vs no exposure	-0.01	0.7	0.92
	Monthly caffeine exposure vs no exposure	0.003	0.92	0.92
	Daily caffeine exposure vs weekly exposure	0.01	0.82	0.92
	Daily caffeine exposure vs monthly exposure	-0.01	0.82	0.92
	Weekly caffeine exposure vs monthly exposure	-0.01	0.67	0.92
	Any caffeine exposure vs no exposure	-0.03	0.46	0.92
	Daily caffeine exposure vs all lower exposure	0.04	0.38	0.92
**Social per CBCL**	Daily caffeine exposure vs no exposure	-0.02	0.45	0.68
	Weekly caffeine exposure vs no exposure	-0.01	0.77	0.77
	Monthly caffeine exposure vs no exposure	-0.04	0.13	0.6
	Daily caffeine exposure vs weekly exposure	-0.01	0.69	0.77

	Daily caffeine exposure vs monthly exposure	0.02	0.45	0.68
	Weekly caffeine exposure vs monthly exposure	0.03	0.28	0.63
	Any caffeine exposure vs no exposure	-0.07	0.11	0.6
	Daily caffeine exposure vs all lower exposure	0.02	0.63	0.77
**Body mass index**	Daily caffeine exposure vs no exposure	0.08	**2.02E-03**	**0.02**
	Weekly caffeine exposure vs no exposure	0.06	**0.049**	0.13
	Monthly caffeine exposure vs no exposure	-0.004	0.91	0.91
	Daily caffeine exposure vs weekly exposure	0.03	0.36	0.48
	Daily caffeine exposure vs monthly exposure	0.09	**7.99E-03**	**0.03**
	Weekly caffeine exposure vs monthly exposure	0.06	0.07	0.14
	Any caffeine exposure vs no exposure	0.16	0.11	0.18

	Daily caffeine exposure vs all lower exposure	0.07	0.63	0.72
**Total sleep problems**	Daily caffeine exposure vs no exposure	0.04	0.12	0.25
	Weekly caffeine exposure vs no exposure	0.06	**0.02**	0.1
	Monthly caffeine exposure vs no exposure	0.04	0.13	0.25
	Daily caffeine exposure vs weekly exposure	-0.02	0.47	0.7
	Daily caffeine exposure vs monthly exposure	-0.002	0.95	0.95
	Weekly caffeine exposure vs monthly exposure	0.02	0.54	0.7
	Any caffeine exposure vs no exposure	0.24	0.14	0.25
	Daily caffeine exposure vs all lower exposure	-0.02	0.9	0.95

Table S4 Note. Linear mixed-effects models were used to analyze the associations between prenatal caffeine exposure and each outcome, nesting data by research site and family identification. Psychotic-like experiences were assessed with the Prodromal Questionnaire Brief-Report Child Version and sleep problems from the Parent Sleep Disturbance Scale for Children. The β coefficients are standardized.

FDR multiple testing correction was conducted across contrasts for each outcome. FDR correction is further explained in the **Methods**.

Due to high missingness of anthropometric data at follow-up waves, BMI was only analyzed as an outcome at the baseline wave.
