# Supplementary material for: Associations between prenatal caffeine exposure and child development: Longitudinal results from the Adolescent Brain Cognitive Development (ABCD) Study
Source: medRxiv. 2024 Jun 19:2024.06.18.24309117. Preprint. [Version 1] doi: 10.1101/2024.06.18.24309117 (PMC11213099; doi:10.1101/2024.06.18.24309117)
Supplement: Supplement 9 [file media-9.pdf]

Table S5. Omnibus Results When Excluding Children With Non-Prevalent Substance Use

**Outcome (n = 8969)**	χ^2	***P* value**	**FDR-corrected P value**
Psychotic-like experiences	5.82	0.12	0.32
Internalizing per CBCL	2.9	0.41	0.63
Externalizing per CBCL	9.15	**0.03**	0.12
Attention per CBCL	1.88	0.6	0.69
Thought per CBCL	0.36	0.95	0.95
Social per CBCL	2.55	0.47	0.63
Body mass index	12.1	**7.04E-03**	0.06
Total sleep problems	5.1	0.17	0.34

Table S5 Note. Excluded children who reported using marijuana (n=72), using substances other than alcohol and tobacco (i.e., bath salts, n=2; "sniff" to "get high," n=9; inhalants, n=14; amphetamines not as prescribed, n=9; tranquilizers/anxiolytics/sedatives not as prescribed, n=11; pain medication not as prescribed, n=4; cough medication to "get high," n=8), or having a full drink of alcohol (n=123) or more than a puff of tobacco (n=36), or screened positive for substances based on hair toxicology (methamphetamines, n=11; THC, n=95; EtG, n=37; cotinine, n=9; Adderall, n=82). A total of 409 individuals were excluded for these analyses (Numbers do not sum as some children were positive across multiple indices). PLEs = Psychotic-Like Experiences. CBCL = Child Behavior Checklist. Due to high missingness of anthropometric data at follow-up waves, BMI was only analyzed as an outcome at the baseline wave.

Table S6. Omnibus Results When Excluding Children Prenatally Exposed to Illicit Substances Other Than Marijuana

**Outcome (n = 8969)**	χ^2	***P* value**	**FDR-corrected P value**
Psychotic-like experiences	7.1	0.07	0.19
Internalizing per CBCL	3.07	0.38	0.51
Externalizing per CBCL	10.02	**0.02**	0.08
Attention per CBCL	3.04	0.39	0.51
Thought per CBCL	0.17	0.98	0.98
Social per CBCL	2.63	0.45	0.51
Body mass index	12.31	**6.4E-03**	0.05
Total sleep problems	6.13	0.11	0.22

Table S6 Note. Children who were exposed to other illicit substances prenatally (i.e., cocaine or crack, n=51; heroin or morphine, n=19; oxycontin, n=25; other, n=33). A total of 102 individuals were excluded (numbers do not sum as some mothers were positive across multiple indices). PLEs

= Psychotic-Like Experiences. CBCL = Child Behavior Checklist. Due to high missingness of anthropometric data at follow-up waves, BMI was only analyzed as an outcome at the baseline wave.

Table S7. Omnibus Results When Excluding Children Born at Extreme Prematurity

**Outcome (n = 8969)**	χ^2	***P*** **value**	**FDR-corrected P value**
Psychotic-like experiences	7.38	0.06	0.16
Internalizing per CBCL	2.76	0.43	0.5
Externalizing per CBCL	10.38	**0.02**	0.08
Attention per CBCL	2.71	0.44	0.5
Thought per CBCL	0.2	0.98	0.98
Social per CBCL	2.86	0.41	0.5
Body mass index	12.48	**5.9E-03**	**0.047**
Total sleep problems	5.27	0.15	0.3

Table S7 Notes. Children born more than 8 weeks premature (i.e., less than 32 weeks' gestation; n=149) were excluded. PLEs = Psychotic-Like Experiences. CBCL = Child Behavior Checklist. Due to high missingness of anthropometric data at follow-up waves, BMI was only analyzed as an outcome at the baseline wave.

Table S8. Omnibus Results When Excluding Children Who Had a Non-Biological Mother Report as the Parent/Caregiver Respondent

**Outcome (n = 8969)**	χ^2	***P*** **value**	**FDR-corrected P value**
Psychotic-like experiences	5.91	0.12	0.32
Internalizing per CBCL	3.78	0.29	0.46
Externalizing per CBCL	7.09	0.07	0.28
Attention per CBCL	1.32	0.73	0.77
Thought per CBCL	1.12	0.77	0.77
Social per CBCL	3.06	0.38	0.51
Body mass index	10.2	**0.02**	0.16
Total sleep problems	4.00	0.26	0.46

Table S8 Notes. Children whose parent/caregiver respondent was not their biological mother were excluded (n=1,617). PLEs = Psychotic-Like Experiences. CBCL = Child Behavior Checklist. Due to high missingness of anthropometric data at follow-up waves, BMI was only analyzed as an outcome at the baseline wave.
